# Adipogenesis Inhibitory Activity of Hypersampsone P from *Hypericum subsessile*

**DOI:** 10.1007/s13659-020-00245-1

**Published:** 2020-05-23

**Authors:** Hui-Min Zhou, Yan-Song Ye, Na-Na Jiang, Rong-Fang Mu, Qian Wang, Jing Hu, Xia Liu, Wan-Ying Qin, Gang Xu, Wen-Yong Xiong

**Affiliations:** 1 State Key Laboratory of Phytochemistry and Plant Resources in West China, Kunming Institute of Botany, Chinese Academy of Sciences, Kunming, 650201 Yunnan People’s Republic of China; 2 University of Chinese Academy of Sciences, Beijing, 100049 People’s Republic of China; 3 Department of Pharmacy, Chongqing Traditional Chinese Medicine Hospital, Chongqing, 400021 China

**Keywords:** Adipogenesis, LKB1, AMPK, Hypericum subsessile, Hypersampsone P

## Abstract

**Abstract:**

Adamantane polycyclic polyprenylated acylphloroglucinols (PPAPs) with caged architecture, a special class of hybrid natural products, is specifically rich in the plant family Guttiferae, especially *Hypericum* or *Garcinia* genus. Hypersampsone P is one of Adamantane PPAPs compounds extracted from *Hypericum subsessile.* Here we have chosen, screened ten PPAPs and identified one of them showed an activity in inhibiting of adipocytes differentiation. Particularly, the compound, hypersampsone P, blunted the adipocyte differentiation dose-dependently. Moreover, hypersampsone P down-regulated the expressions of several key regulators for adipogenesis, including PPARγ and FABP4. The treatment of cells at the early stage of adipogenesis by hypersampsone P induced the greatest blunting of adipocyte differentiation and the effect might be involved in the LKB1-AMPK signaling pathway.

**Graphic Abstract:**

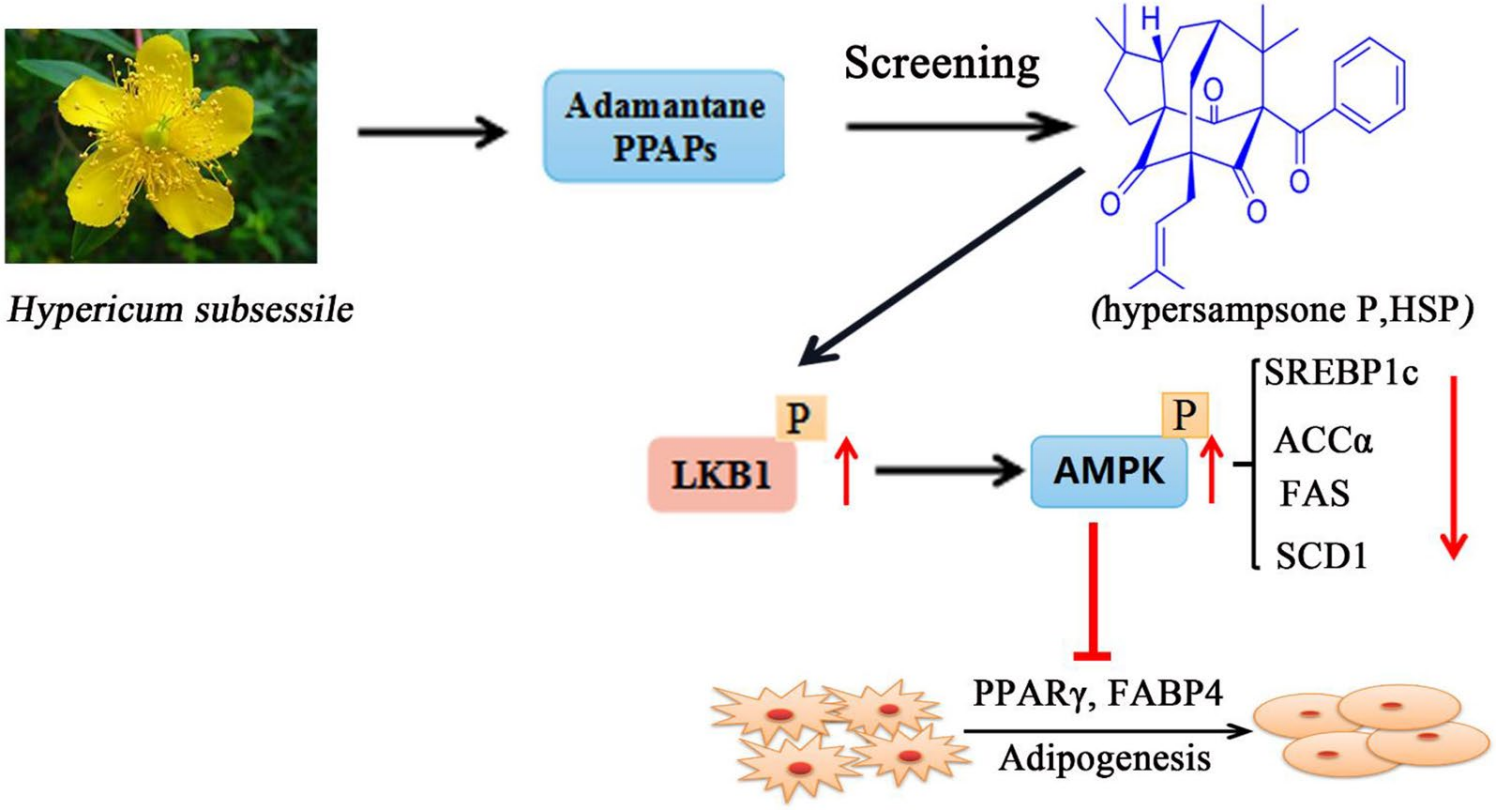

## Introduction

The overweight and obesity is an important origin for metabolic diseases, including high cholesterol, hyperlipidemia, diabetes and hypertension [1]. Prevalence of obesity and overweight has risen substantially in the past three decades, and more and more children became obese [2, 3]. The common therapies of obesity are surgery and pharmacotherapy [4]. Surgical treatment for severely obese patients has certain risks and postoperative side effects [5]. The drugs for obesity treatment are also accompanied by cardiovascular disease, constipation, gastrointestinal adverse reactions and other side effects while alleviating obesity [6, 7].

The obesity is mainly the result of over-nutrition on adipocyte tissue and the abnormal development of adipocytes [8], known as adipocyte differentiation or adipogenesis, and the disease is related to unbalanced energy homeostasis [9]. Therefore, the regulation of adipogenesisis is significant for obesity prevention and treatment [10]. Adipogenesis is regulated by complex network of transcription factors that coordinates the expression of many phenotypic proteins in adipocyte differentiation [11]. Peroxisome proliferator activated receptor gamma (PPARγ) plays an essential role in white adipose tissue, which is involved in adipogenesis and lipid metabolism [12, 13]. And fatty acid binding protein 4 (FABP4) is a marker protein for adipocyte differentiation, regulated by PPARγ [14]. These transcription factors regulators are regulated by multiple signaling pathways include AMPK signaling pathway. AMP-activated protein kinase (AMPK), a serine/threonine protein kinase that is widely expressed in eukaryotes [15], is a central regulator for obesity and energy homeostasis [16, 17]. AMPK is regulated by LKB1 (Liver kinase B1), which performs its biological functions through phosphorylation at sites such as S428 site [18]. In skeleton muscles, AMPK is activated in response to exercise and its activators has been proved as a potential target for obesity and T2DM [19, 20]. There is increasing evidence that AMPK inhibits the expression of PPAR and FABP4 and blunts adipogenesis in adipocytes [20–22]. It was reported that Euphorbiasteroid blunted the differentiation of human preadipocytes by enhancing the phosphorylation of AMPK for down-regulating PPARγ and fatty acid biosynthesis include sterol regulatory element-binding transcription factor 1 (SREBP1c), fatty acid synthase (FAS), acetyl-CoA carboxylase (ACC)α and stearoyl-CoA desaturase (SCD) [23]. Thus, natural products that affect the AMPK signaling pathway may prevent obesity.

Polycyclic polyprenylated acylphloroglucinols (PPAPs) are characteristic secondary metabolites in the plants of *Hypericum* genus, yet the little is known for its bioactivities [24]. Only several PPAPs compounds from *Hypericum patulum*, has been showed inhibit T-type calcium channels [25]. Adamantane polycyclic polyprenylated acylphloroglucinols with caged architecture, a special class of hybrid natural products, is specifically rich in the plant family Guttiferae, especially *Hypericum* genus [26]. However, the bioactivity of Hypersampsone P, one of Adamantane PPAPs compounds extracted from *Hypericum subsessile* is still unknown [27]. Here we have screened a serial of PPAPs for their potential effects in adipocyte differentiation. Among the PPAPs, we identified the hypersampsone P shows a promising activity in regulating of adipogenesis and explored its potential mechanism in the effect. The result supported a novel function of the compound in regulation of adipocyte differentiation, indicating it is a potential candidate for treatment of obesity in near future.

## Results

### Screening the Effects of PPAPs on Adipocyte Differentiation

**Fig. 1:**
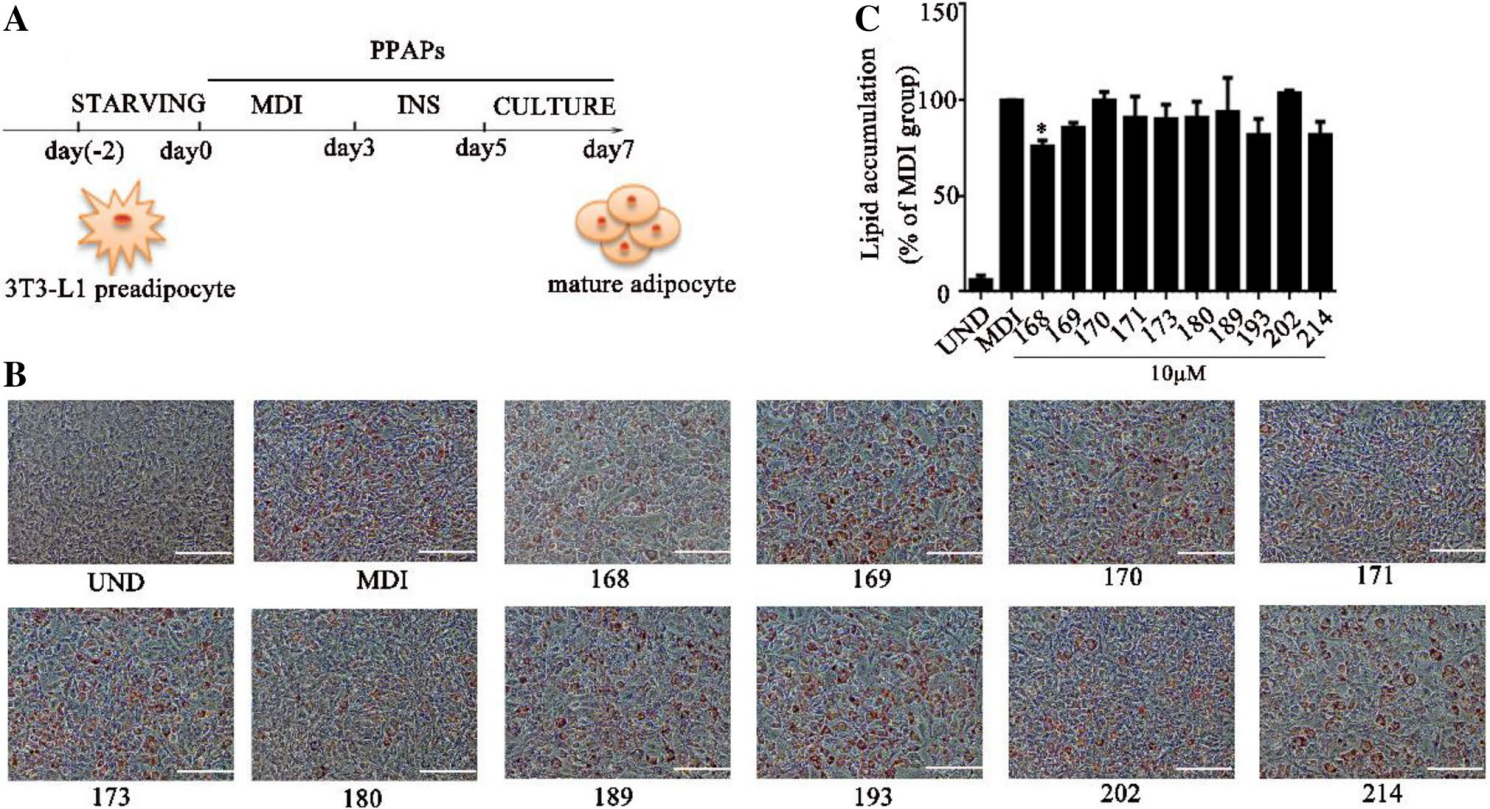
Screening of PPAPs in regulating of adipocyte differentiation. **a** Schematic diagram of inducing 3T3-L1 adipocyte differentiation. **b** Representative images of the cells treated with ten of PPAPs (10 µM) and stained by Oil Red O. Scale bars, 100 µM. **c** Normalized lipid content by measuring Oil Red O intensity of the cells from **b**. Date are showed as mean ± SD of three independent experiments and normalized to the value of MDI. *p < 0.05 versus MDI group

Here we have screened ten PPAPs by a classic cell model of adipocyte differentiation (Fig. 1a). Surprisingly, one of them (compounds 168) blunted the process of adipogenesis by measuring the changes of lipid content in the cells after fully inducing of adipogenesis (Fig. 1b).

**Fig. 2:**
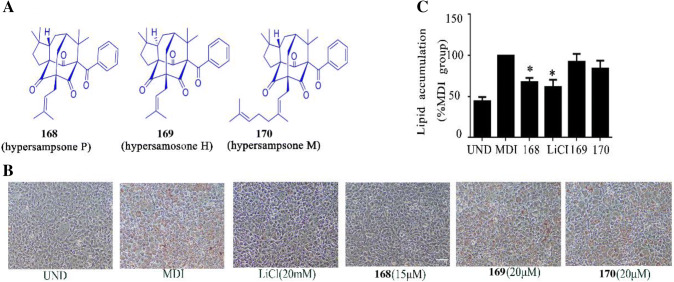
Specificity of the compound with activity in adipogenesis. **a** Structures of the three compounds **168** (hypersampsone P), **169** (hypersampsone H) and **170** (hypersampsone M) with the most similarity. **b** Representative images of 3T3-L1 adipocytes treated with the three compounds and stained by Oil Red O. Scale bars, 100 µM. **c** Normalized lipid content of the cells from **b**. *p < 0.05 versus MDI group

Then we choose three compounds, among the ten compounds, with the most similar chemical structure to insure the effect with the unique structure for the bioactivity. As expected, compound **168** (hypersampsone P) was able to inhibit 3T3-L1 differentiation to the degree as that of the positive control-LiCl, whereas higher concentrations of **169** (hypersampsone H) and **170** (hypersampsone M) did not affect the process (Fig. 2b, c). In combination with the chemical structures, the hydrogen on the 5-position carbon of compound **169** is chiral, and the 28-position carbon of compound **170** has geranyl group substitution (Fig. 2a). The structure–activity relationships of these three compounds indicated that the hydrogen of 5-position carbon was necessary for the activity. Moreover, if there is the structure on the 28th site of geranyl group, the activity of hypersampsone P on adipogenesis can be reduced. These observations are meant to aid in the understanding of the administration of hypersampsone P in terms of its potential prevention or treatment of obesity.

These results support the effect hypersampsone P on adipocyte differentiation and the unique structure of the chirality of hydrogen on the fifth site and the absence of geranyl group on the 28th site of hypersampsone P.

### Hypersampsones P Inhibits Adipogenesis Dose-Dependently

**Fig. 3:**
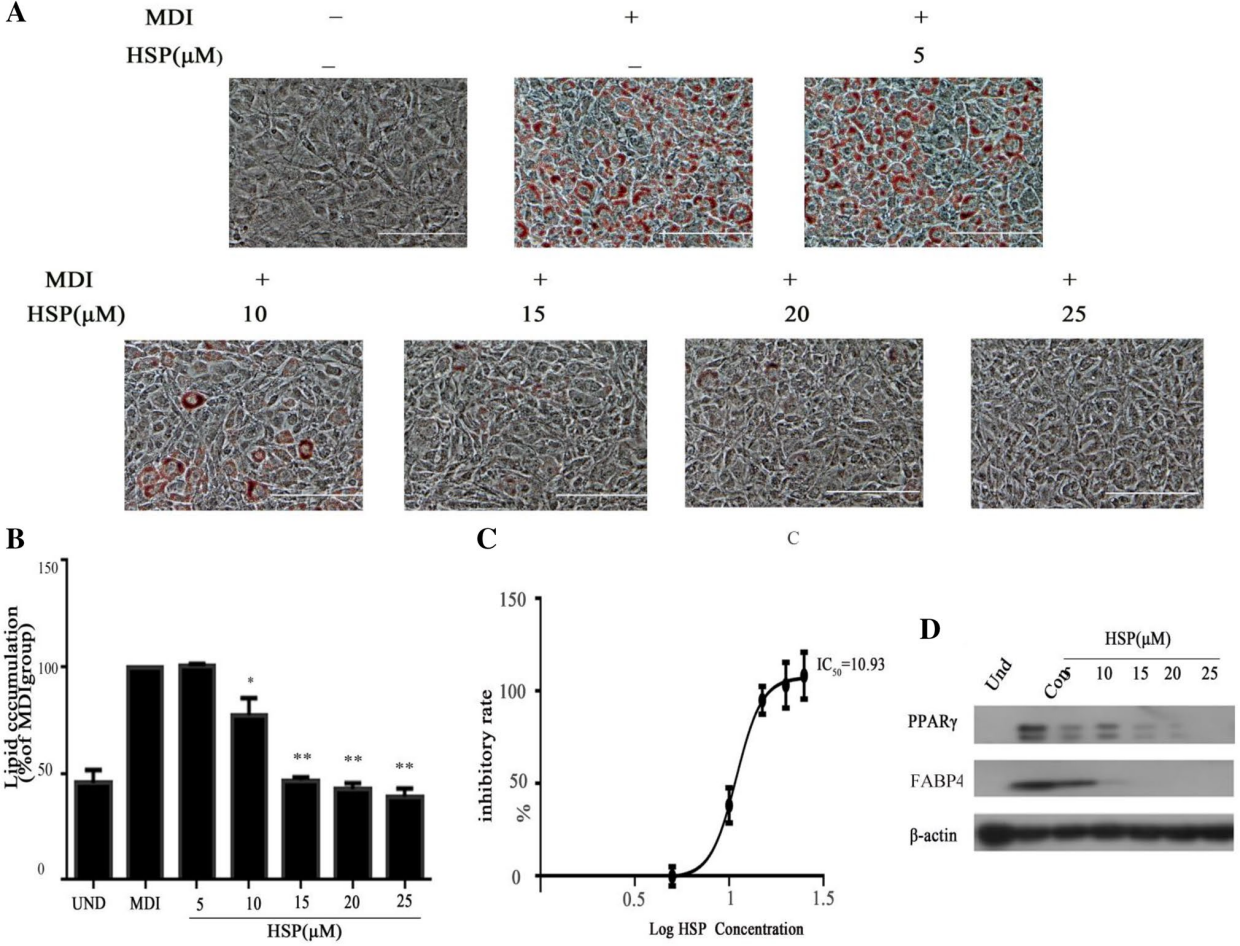
Hypersampsone P inhibited adipogenesis dose-dependently. **a** Representative images of 3T3-L1 adipocytes treated with a series of doses of hypersampsone P (5 to 25 µM) and stained by Oil Red O afterwards. Scale bars, 100 µM. **b** Normalized lipid content by measuring Oil Red O intensity of the cells from **a**. **c** The dose–response curve of hypersampsone P on the inhibition of adipocyte differentiation. **d** Expressions of key regulators for adipogenesis, PPARγ and FABP4. Date are showed as mean ± SD of three independent experiments. *p < 0.05, **p < 0.01 versus MDI group

We next elucidated the specificity of hypersampsone P in regulation of the adipogenesis. Similarly, we treated cells with a series of concentrations in the range of 5–25 μM, and measured the lipid contents at the end of inducing adipogenesis. As shown, hypersampsone P blunted the lipid content in cells after inducing of the adipogenesis starting after 5 μM and almost reached a maxim inhibition about 20 μM (Fig. 3a, b), supporting the effect was dose-dependent (Fig. 3c).

In consistent to the effect above, the expressions of several differentiation markers during adipogenesis, PPARγ and FABP4, were down-regulated, proving the effect of hypersampsone P on adipogenesis (Fig. 3d).

### Hypersamsone P Blunts the Adipogenesis at the Early Stage

**Fig. 4:**
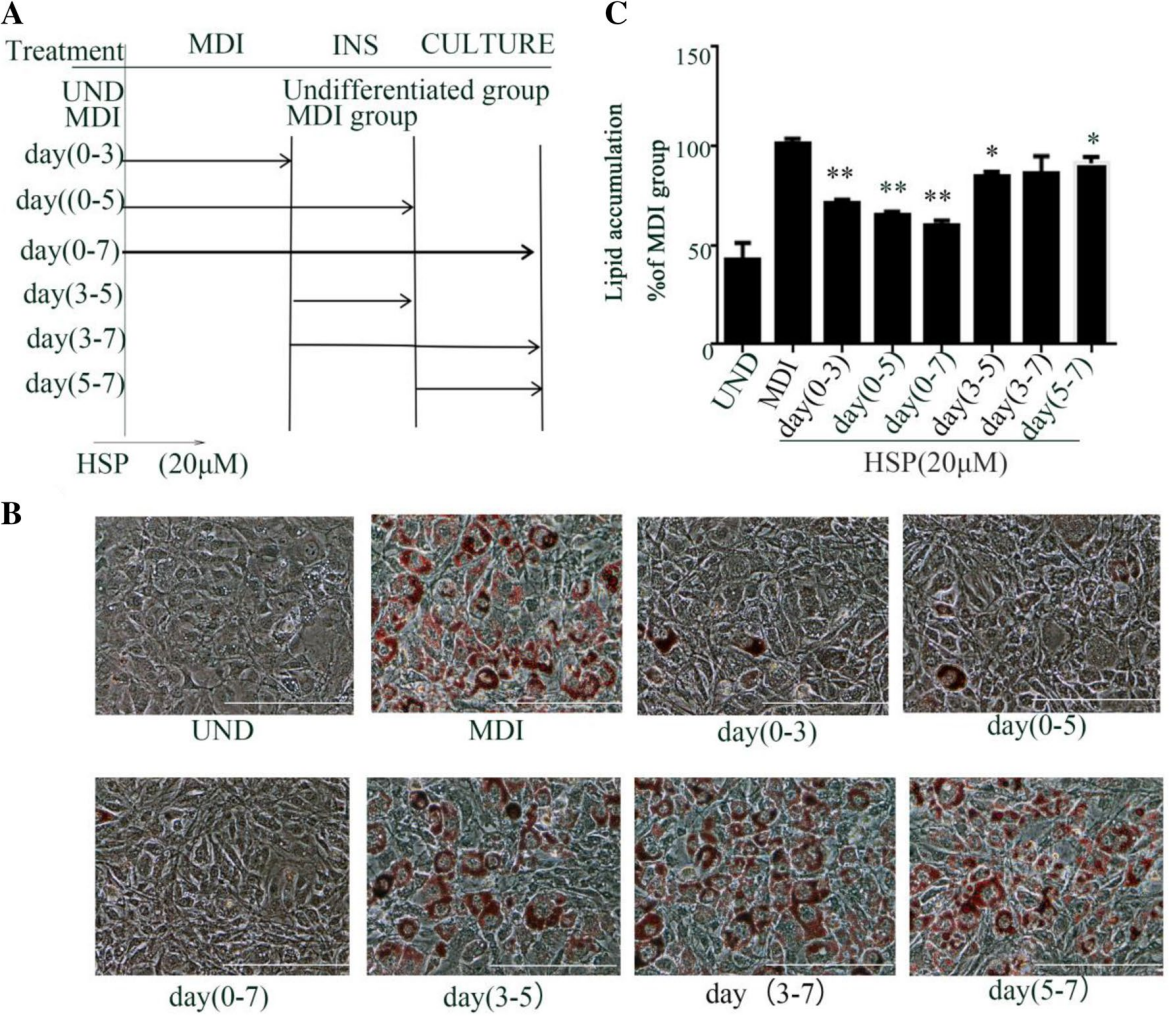
Hypersampsone P blunted adipocyte differentiation at the early-stage. **a** Workflow of cells treated hypersampsone P during the different periods of adipocyte differentiation. **b** Representative images of 3T3-L1 adipocytes treated by hypersampsone P at the different stage of differentiation as shown in **a**. Scale bars, 100 μM. **c** Normalized lipids content by measuring intensity of Oil Red O of the cells from **b**. Date are showed as mean ± SD of three independent experiments. *p < 0.05, **p < 0.01 versus MDI group

The adipocyte differentiation is consist of several stages. To elucidate how hypersampsone P affect the adipogenesis, we treated the cells at different stages of adipogenesis (Fig. 4a) and measured the lipid content (Fig. 4b). Not surprisingly, the maxim inhibition of hypersampsone P on the differentiation were observed in the cells incubated by the compound at day 0–3, day0-5, or day 0–7, whereas the treatments at the day 3–5, day 3–7 or day 5–7 with hypersampsone P only inhibited the differentiation mildly (Fig. 4c), indicating hypersampsone P inhibits adipogenesis at the early stage of differentiation.

### Hypersampsone P Upregulated the LKB1-AMPK Signaling Pathway

**Fig. 5:**
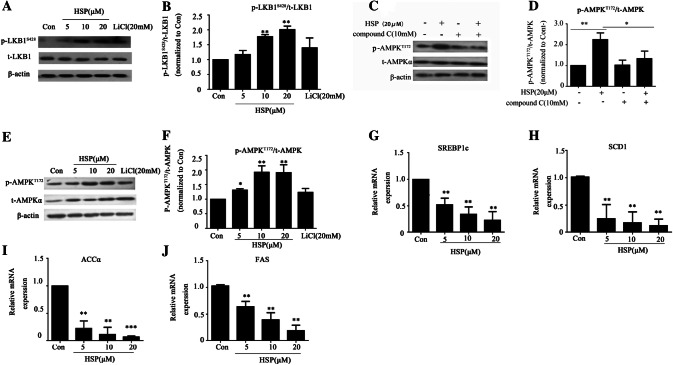
Hypersampsone P regulated LKB1-AMPK signaling. **a**, **b** The expression of AMPK regulator protein, t-LKB1 and p-LKB1 Western blotting and quantitatively analyzed. **c, d** Protein levels of p-AMPK^T172^ treated by hypersampsone P and with/without an AMPK inhibitor, compound C and quantitatively analyzed. **e**, **f** Protein expression of t-AMPK, p-AMPK of the cells treated by (5–20 μM) hypersampsone P and quantitatively analyzed. **g**–**j** The expression of AMPK downstream genes, (SREBP1c, FAS, ACCɑ, SCD1) by real-time PCR. Date are showed as mean ± SD of three independent experiments. *p < 0.05, **p < 0.01, ***p < 0.001 versus Con group

During the early stage of adipocyte differentiation, LKB1-AMPK signaling pathway plays a key role. Therefore we collected samples on the third day after induction and elucidated the phosphorylation of the LKB1^S428^. Interestingly, hypersampsone P did induce an increasing of the phosphorylation of LKB1^S428^ (Fig. 5a, b). Moreover, under the circumstances of the phosphorylation of AMPK^T172^ was blocked by an AMPK inhibitor-compound C, the upregulated AMPK phosphorylation by hypersampsone P was abolished, supporting that 20 μM hypersampsone P affected the AMPK signaling pathway (Fig. 5c, d). We detected the phosphorylation of the AMPK^T172^ treated by hypersampsone P (5–20 μM). It also up-regulated by hypersampsone P dose-dependently (Fig. 5e, f). In addition, hypersampsone P down-regulated the gene expression of SREBP1c, ACCα, SCD1, FAS which all belong to the downstream of AMPK (Fig. 5g–j).

## Dissussion

In this study, we demonstrated the activity of hypersampsone P in adipocytes differentiation. It indicated a potential development of the compound, as a new functional structure and a potential probe for exploring mechanism in chemistry biology.

First of all, we proposed the function of hypersampsone P in adipogenesis is most likely rely on the chirality of hydrogen on the fifth site and the absence of geranyl group on the 28th site, because the other two compounds similar to hypersampsone P, hypersampsone H and hypersampsone M, did not affect the process of adipogenesis (Fig. 2), providing an evidence of the unique structure of the compound on the effect.

Regarding of the mechanism, our data demonstrated that hypersampsone P affected the process of adipogenesis remarkably as long as we treated cells during the first 3-day inducing of the differentiation. Yet, the effects were significantly weaken if the differentiation were treated by hypersampsone P after the 3-day inducing (Fig. 4). Furthermore, we hypothesis that hypersampsone P might affect the LKB1, AMPK phosphorylation, along with the downstream SREBP1c, ACCα, SCD1, FAS’s gene expression during the early stage of the differentiation (Fig. 5). LKB1 and AMPK are central sensors and regulators for cell metabolism and energy homeostasis. The effect of hypersampsone P on LKB1-AMPK pathway highlights a reasonable mechanism for the effect. However, lacking of enough amount of hypersampsone P limited our research in cell study only, in addition, how hypersampsone P targets to the LKB1-AMPK signaling pathway is still unknown.

Nevertheless, our results clearly demonstrated hypersampsone P’s function in regulation of adipocytes differentiation, which is related to the overweight and obesity. It might be an alternative candidate as parent-structure for developing drug candidates for treatment of obesity in future.

## Materials and Methods

### 3T3-L1 Culture and Differentiation

3T3-L1 mouse preadipocytes were purchased from the American Type Culture Collection (ATCC, Manassas, VA, USA). Cells were cultured in DMEM supplemented with 10% CS. Two days after confluence (day 0), cells were induced for differentiation with DMEM supplemented with 10% FBS, IBMX, DEX and insulin (designated hereafter as MDI) as previously described [28]. On day 3, the medium was changed to medium containing 10% FBS and 1 µg/mL insulin for 2 days (day 5), and then insulin was removed from the 10% FBS-DMEM for another 2 days. The cells were fully differentiated into mature adipocytes on day 7.

### Oil Red O Staining

After removing the culture medium, 3T3-L1 cells were washed two times with PBS and subsequently fixed in 10% formaldehyde overnight at room temperature. After fixing, the cells were washed with water twice and one time with 60% isopropanol then the cells were stained with Oil Red O working solution for 10 min, washed with water, and then photos were taken under microscopy. To quantify lipids content, stained 3T3-L1 cells were washed with 100% isopropanol and the absorbance was measured at 492 nm by a microplate reader (Perkin Elmer Envision Multilabel reader). The absorbance in MDI group was standardized to 100%.

### RNA Isolation and Real-Time PCR

RNA was extracted from 3T3-L1 cells using Trizol reagent (SANGONG BIOTECH), and cDNA was synthesized using cDNA synthesis kits using total RNA (2 μg) (Applied Biological Materials Inc). Real-time PCR cDNA gene expression was detected using the SYBR Green Master kit and a spectrofluorometric thermal cycler (Applied Biosystems).

**Table:** 

Gene	Forward primer	Reverse primer
SREBP1c	ACAGACAAACTGCCCATCCA	GCAAGAAGCGGATGTAGTCG
ACCɑ	TGAGAAGGTTCTTATCGCCAACA	TTCATAAGACCACCGACGA
SCD1	ATGGATATCGCCCCTACGAC	GATGTGCCAGCGGTACTCAC
FAS	ACCCTGACCCAGAATACCAAG	GTCAACAACCATAGGCGATTT
β-actin	CACCCCAGCCATGTACGT	GTCCAGACGCAGGATGGC

### Western Blotting Analysis

Cells were washed twice with ice-cold PBS and subsequently lysed in equal volumes of cell lysis buffer containing protein inhibitor cocktail (Sigma) for 30 min. Protein concentration was measured by BCA protein assay kit (Pierce). The lysates mixed with a certain amount of 5 × loading buffer and boiled at 100 ℃ for 10 min. The protein was separated by 10% SDS-PAGE and transferred to PVDF membrane, blocked in 5% non-fat dried milk dissolved in PBS-T for 1 h and immunoblotted with antibodies specific for PPARγ, FABP4, t-AMPKα, P-AMPK^T172^, t-ACC, P-ACC^S79^ and β-actin. Finally, the immunoblots were quantified using the Metamorph software and expressed as a ratio of phosphorylated to total protein or total protein to β-actin.
